# Endoscopic and Surgical Treatment in Early Gastric Cancer: The Gray Zone in Treatment Decision-Making from the Perspectives of Endoscopists

**DOI:** 10.3390/cancers17040602

**Published:** 2025-02-10

**Authors:** Jun Yong Bae, Chang Beom Ryu, Moon Sung Lee, Stavros Dimitriadis

**Affiliations:** 1 Digestive Disease Center, Department of Internal Medicine, Seoul Medical Center, Seoul 02053, Republic of Korea; echosys05@gmail.com; 2 Digestive Disease Center and Research Institute, Department of Internal Medicine, SoonChunHyang University School of Medicine, Bucheon 14584, Republic of Korea; mslee@schmc.ac.kr; 3 Gastroenterology Private Practice, Egnatia 106, 54622 Thessaloniki, Greece; dimitriadis.stavros@yahoo.com

**Keywords:** early gastric cancer, gray zone, endoscopic treatment, surgical treatment, endoscopic submucosal dissection

## Abstract

To treat early gastric cancer, one must choose between endoscopic treatment and surgical treatment. Endoscopic treatment has been developing significantly since the late 1990s and has made great progress up to the present. However, many patients with early gastric cancer still undergo unnecessary surgery or endoscopic procedures. This is due to the existence of a “gray zone” of ambiguities between endoscopic and surgical treatment. These ambiguities arise because the important factors in determining the treatment for early gastric cancer can only be fully understood after endoscopic or surgical resection or because of discrepancies between the factors identifiable before treatment and those identifiable after treatment. This article aims to explore these ambiguous factors and discuss methods and efforts to reduce them.

## Simple Summary

When determining the treatment for early gastric cancer, several factors must be considered to decide between endoscopic treatment and surgery. There are limitations in the information available before treatment, making it difficult to predict the final outcome. This leads to the occurrence of the “gray zone”. This article examines these factors and highlights issues related to diagnosis, endoscopic treatment, and tissue-handling processes from the perspective of endoscopists. In particular, this article points out issues arising from differences in the anatomical location and handling processes of the obtained tissue between surgery and endoscopic procedures. Since the handling processes of tissue in surgery and endoscopy are inherently different, this article emphasizes that the endoscopic treatment indications derived from surgical outcome analyses should be further developed based on the results from endoscopic treatment.

## 1. Introduction

Endoscopic treatment for early gastric cancer (EGC) began in the 1990s, driven by the need for less invasive but equally effective therapeutic options. To ensure safe and effective treatment, a comprehensive guideline was developed based on clinical outcomes from surgical treatments. This guideline initially established the absolute indication for endoscopic treatment by analyzing factors such as tumor size, histologic differentiation, and ulcer presence, which are closely associated with the risk of lymph node metastasis [1].

With advancements in endoscopic tools and techniques, as well as growing expertise among endoscopists, the treatment of larger and more complex lesions has become feasible. Endoscopic submucosal dissection (ESD), using specialized endoscopic knives, has replaced endoscopic mucosal resection (EMR) as the preferred method for treating EGC, offering significantly higher complete resection rates. Despite these advancements, outcomes of endoscopic treatment for EGC remained suboptimal, especially for larger and more complex lesions. This led to the development of the expanded indication for endoscopic treatment, based again on the patients’ surgical outcomes [2].

The categorization, as outlined in the Japanese Gastric Cancer Treatment Guidelines from the 1st to the 6th editions, is summarized in Table 1 [3,4,5,6,7,8]. This table presents the indications for endoscopic treatment according to the guidelines, along with the changes in these indications and terminology over the years.

In a meta-analysis comparing ESD and surgery for EGC with absolute and expanded indications, the 5-year overall survival, disease-specific survival, and disease-free survival rates were found to be similar for both treatments. However, the rates of recurrence, synchronous cancers, and metachronous cancers were higher with endoscopic treatment compared to surgery [9]. The evolution of endoscopic techniques has expanded the indications for ESD, providing a safe and effective alternative to surgery.

**Figure 1:**
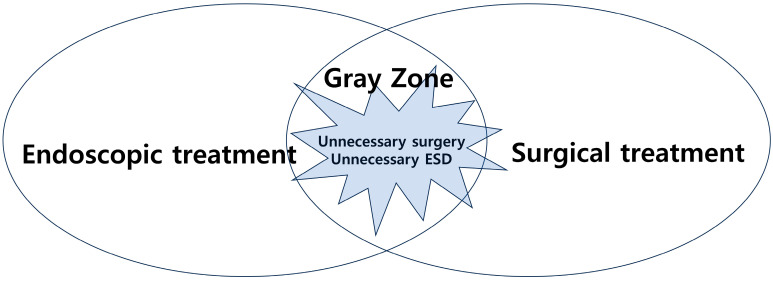
The concept of the gray zone between endoscopic treatment and surgical treatment.

However, there are still ambiguities in the application of endoscopic treatment and surgery when determining the appropriate approach for EGC. In some cases, patients undergo unnecessary surgery or endoscopic treatment, highlighting the importance of reducing this gray zone (Figure 1). At this point, the question arises: where does the definite boundary lie within the gray zone between endoscopic treatment and surgery? This issue persisted even during the transition from absolute indication to expanded indication in the past. The goal of this article is to explore the gray zone and the factors that influence it.

## 2. Factors Affecting Endoscopic Treatment

1. Depth of invasion;
2. Histologic ulceration;
3. Histologic differentiation;
4. Tumor diameter (size);
5. Evaluation of margin;
6. Lymphovascular invasion.

The six factors mentioned above serve as criteria for determining the feasibility of endoscopic treatment. When endoscopic treatment is performed, these factors also determine the success of the procedure or the need for additional surgery. However, it is important to consider whether the observations and measurements of these criteria can vary. Assessing the accuracy and reliability of these criteria is essential, especially considering that their interpretation may differ based on the clinical context or the stance of the endoscopist or pathologist.

### 2.1. Depth of Invasion

The stomach is anatomically divided into five distinct layers, each with clear histologic distinction. For example, it is usually easy to distinguish between an invasion of the mucosa and submucosa or between the submucosa and the muscularis propria.

Accurate and precise sectioning of histologic tissue is important for assessing the depth of tumor invasion. Creating dense sections of approximately 2–3 mm intervals allows for accuracy and precision [8]. With such sectioning, it becomes clear which specific layer the tumor has invaded [10].

Challenges arise in more detailed assessments. In particular, it can be difficult to accurately measure and distinguish the degree of invasion in the submucosal (SM) layer, especially when determining whether the invasion is within 500 μm or classifying it as SM1, SM2, or SM3. The current 500-micrometer (SM1) standard is based on specimens resected through surgery, which include all five layers of the stomach. Until now, there has been no better parameter than this. However, the ESD flap comprises only the gastric mucosa and the SM layer, which is the loosest layer and can be significantly affected by handling during and after the procedure. Therefore, it is questionable whether the 500-micrometer measurement in surgical tissue is applicable to the ESD specimen. If a significant portion of the SM layer obtained through endoscopic procedures is below 500 μm or if there is significant damage, such as a large hole in the flap, it can create significant difficulties in accurate evaluation. Moreover, the flaps of the cardia, fundus, and upper body (thinner flaps) and the flaps of the antrum (thicker flaps) have different thicknesses, but the current indication does not take this into account. These anatomical differences can further complicate the assessment, as the varying thickness of flaps between different stomach regions could influence the accuracy of depth measurement and subsequent diagnosis.

To accurately assess the depth of invasion, efforts should be made to minimize thermal injury to the SM layer during ESD treatment. To minimize damage to the flap and ensure precise treatment, careful observation of the layer during endoscopic procedures is also essential. Additionally, direct coagulation of the flap due to bleeding from the flap’s vessels should be avoided to preserve the integrity of the SM layer and facilitate accurate evaluation. It is preferable to locate the perforating vessel and control the bleeding rather than directly coagulate the bleeding site of the flap.

### 2.2. Histologic Ulceration

A gastric ulcer is defined as the destruction of the stomach lining extending beyond the muscularis mucosa [11,12]. In the indication of endoscopic treatment, the criteria for ulceration are based on histologic rather than gross (endoscopic) ulceration [8]. However, biopsy tissue alone cannot reliably determine ulceration, and histologic ulceration can only be fully evaluated after endoscopic treatment through a complete specimen [8,13]. Therefore, when determining an indication for endoscopic treatment before the procedure, it is necessary to consider gross ulceration observed during endoscopy.

However, EGC has a lifecycle that includes depressed, ulcerative, and healing phases. The appearance of the lesion varies depending on the time of observation [14]. There may be confusion due to a shallow ulceration or a healing ulcer due to ulcer medications such as proton pump inhibitors (PPIs) seen endoscopically. The impact of PPIs on gastric cancer is still controversial [15]. There is one report on the healing of ulcers in differentiated-type EGC related to PPI use [16] but it remains difficult to fully understand its impact. Additionally, distinguishing true ulceration from biopsy-related artifacts can sometimes be challenging during histologic evaluation. In one study, it was found that the diagnosis of ulcers on endoscopy was overestimated in 38.7% of lesions, and they were particularly observed in the lower third of the stomach [17].

### 2.3. Histologic Differentiation

Histologic differentiation in gastric cancers is determined by the quantitative predominant type of cellular differentiation, typically requiring that more than 50% of the tumor cells exhibit a specific differentiation [5,8]. This criterion can lead to discrepancies between pre-treatment biopsy results and post-treatment specimen analysis after endoscopic treatment, as biopsies may not fully capture tumor heterogeneity [18]. In a comprehensive study investigating differentiated-type EGC, mixed histology was observed in 2.7% of cases, with larger tumor size, mid-third location, and moderate differentiation identified as independent risk factors for mixed histology in forceps biopsies [19]. A meta-analysis of mixed histology and lymph node metastasis showed that both differentiated and undifferentiated-type EGC demonstrated higher lymph node metastasis rates in mixed types compared to pure types [20].

There are limitations of the pre-treatment biopsy in assessing the histologic predominance and mixed histology. As a result, fully understanding the histologic differentiation based solely on pre-treatment endoscopic appearance and biopsy results remains challenging in certain cases.

### 2.4. Tumor Diameter (Size)

The tumor diameter (size) is also an important factor in determining the choice between endoscopic treatment and surgical treatment for EGC. This factor is based on the final diameter (size) measured during histologic evaluation after the endoscopically resected specimen is fixed in formalin [21]. The issue with this measurement is that there are no clear guidelines or criteria for the amount of tension to be applied when pinning and stretching the specimen. Additionally, the specimen may shrink during the formalin fixation process, further complicating the measurement. As mentioned earlier, there are anatomical differences in the thickness of the mucosa and submucosa in the different regions of the stomach.

However, there is no consideration of the differences in the deformation of the specimen depending on the location during the pinning and stretching process and after formalin fixation. The original indications for endoscopic treatment were based on surgically resected histologic specimens, so it makes sense that the final diameter (size) measured in the histologic specimen is used as the standard. Surgical specimens consist of five layers, including a strong proper muscle structure, while endoscopically resected specimens are composed only of the mucosa and submucosa, which are the loosest tissues in the stomach. It is unreasonable to expect the same changes for these two different specimen types. Furthermore, while making treatment decisions based solely on endoscopic observation, it is difficult to predict the final diameter (size) of the specimen that has been endoscopically resected and fixed in formalin.

**Figure 2:**
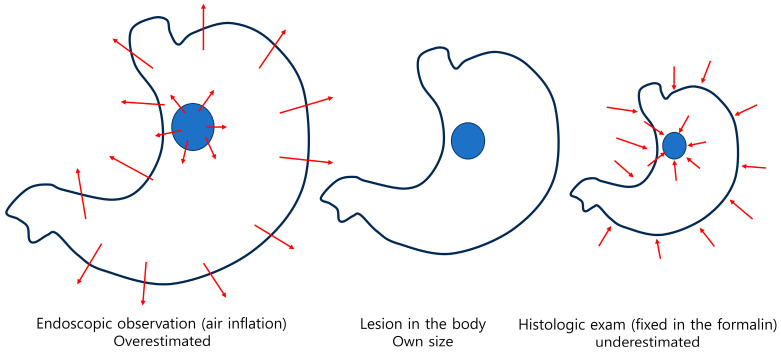
Theoretically considered changes in the surgically resected stomach.

When resected tissue is fixed in formalin, it generally shrinks, although the extent varies depending on the location and composition of the tissue (Figure 2). A study analyzing changes in the thickness and size of fresh and formalin-fixed specimens from laparoscopic sleeve gastrectomy tissues showed notable differences between tissues containing full-thickness layers and those comprising only the mucosa and submucosa (mucosa/submucosa) of the fundus, corpus, and antrum [22]. After formalin fixation, the full-thickness tissues showed a thickness increase of 4.4, 5.6, and 7.2 times and a size reduction of 21.7%, 23.2%, and 25.2% in the antrum, corpus, and fundus, respectively. Meanwhile, the mucosa/submucosa tissues showed a thickness increase of 1.8, 2.5, and 4.6 times and a size reduction of 28.0%, 32.9%, and 35.3% in the antrum, corpus, and fundus, respectively. According to this study, it can be concluded that formalin fixation causes an increase in tissue thickness and a reduction in size, with variations observed depending on the gastric location and type of specimen. A noteworthy aspect of this study is that, unlike the conventional practice of pinning and stretching the margins with multiple pins after endoscopic treatment, the tissue was fixed using only a single pin at its center. This approach was taken to compare changes in thickness and size between full-thickness tissue and mucosa/submucosa tissue.

**Figure 3:**
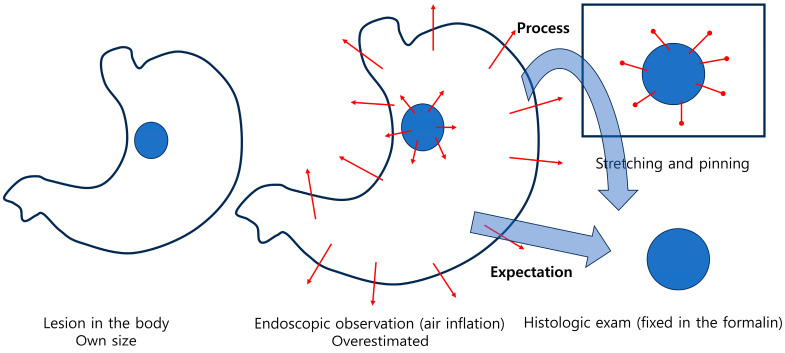
Theoretically considered changes in the endoscopically resected specimen.

Ironically, two studies comparing the size of lesions observed during endoscopy with those in ESD specimens during histologic evaluation after formalin fixation found that the size of the lesions actually increased during histologic evaluation after fixation, contrary to the expected shrinkage during the fixation process [23,24]. This phenomenon may be attributed to the process of pinning and stretching the specimen after endoscopic resection, and then fixing it in formalin (Figure 3). In one study [23], measurement discrepancies based on histologic lesion size were observed as follows: for lesion sizes of 1.0 cm or less, the measurement discrepancy mean was 0.04 cm; for lesion sizes between 1.0 and 2.0 cm, the mean was 0.14 cm; for lesion sizes between 2.0 and 3.0 cm, the mean was 0.31 cm; for lesion sizes between 3.0 and 4.0 cm, the mean was 0.68 cm; and for lesion sizes between 4.0 and 5.0 cm, the mean was 0.80 cm. In another study [24], the overall median size of the lesions was 12.0 mm (IQR 10.0–15.0) during endoscopic observation and 12.0 mm (IQR 7.0–20.0) during histologic evaluation (*p* = 0.367). The overall median size discrepancy was 5.0 mm (IQR 2.0–9.0). When the tumor size exceeded 2 cm, the size observed on endoscopy was underestimated compared to the histologic measurement. Both studies showed that the size of the lesions increased during histologic evaluation compared to endoscopic observation, particularly as the lesions were larger and of undifferentiated-type histology.

### 2.5. Evaluation of Margin

It is important to avoid challenges in assessing margins due to electrical injury during histologic evaluation. During endoscopic procedures, close attention should be taken to prevent electrocautery damage to the margins from marking dots, knives, or other instruments. After the procedure, resected specimens, which tend to curl easily, should be carefully stretched with minimal tension and pinned for proper fixation. Following these steps ensures that margin evaluation can be conducted with minimal ambiguity.

### 2.6. Lymphovascular Invasion

Several studies on needle-based tissue biopsy have raised concerns about cellular contamination. This issue typically arises when the needle directly punctures the tumor, as seen in procedures like liver biopsy or EUS-guided fine needle aspiration/biopsy. Although the likelihood of contamination is very low, it could result in the spread of tumor cells to surrounding tissues or errors in histologic diagnosis [25,26,27]. While no documented cases of metastasis or other complications have been reported from directly puncturing a tumor using an endoscopic injector, the act of puncturing the tumor site for submucosal injection carries a theoretical risk of cellular contamination. There is also a theoretical possibility that these contaminated tumor cells could be introduced into blood vessels or lymphatic tissues. Therefore, during endoscopic procedures, it is essential to avoid directly puncturing the tumor when attempting submucosal injection.

In order to accurately assess lymphovascular invasion, electrocautery damage to the SM layer should be minimized. To minimize damage to the SM layer of the specimen, when bleeding occurs from the flap, it is preferable to locate the perforating vessel and control the bleeding rather than directly coagulating the bleeding site of the flap.

## 3. The Gray Zone Between Endoscopic Treatment and Surgical Treatment

The reason a gray zone arises is that, when deciding on the treatment for EGC, it is necessary to predict factors that can only be definitively determined after endoscopic resection. These factors should be predicted solely using pre-treatment evaluations such as endoscopy, biopsy, computed tomography, and endoscopic ultrasonography. Based on these evaluations, the appropriate treatment strategy should be determined before treatment.

The endoscopic curability (eCura) scoring system [28,29,30] may have a gray zone within the low-risk category (0–1 point). When factors such as ulceration, undifferentiated type, tumor diameter (size) over 3 cm, positive vertical margin, pT1b-SM2 invasion, or vascular invasion are present individually, the likelihood of lymph node metastasis is as low as 2.5%. Additional research and data collection are needed to clarify the prognosis when these factors are present independently.

Table 2 summarizes the distinct zones and gray zones, the actions or steps that influence them, and efforts to minimize these uncertainties. Additionally, based on the authors’ perspectives, the levels of understanding have been categorized into four stages: weak, relatively weak, relatively strong, and strong.

Among these factors, tumor diameter (size) is considered to be an area with high ambiguity. As previously mentioned, differences in processing methods between surgically resected specimens and endoscopically resected specimens contribute to this ambiguity. While size assessments based on surgically resected specimens may underestimate the actual size of the lesion in vivo, the measurements from endoscopically resected specimens may overestimate the size due to the pinning and stretching process during specimen preparation.

In fact, studies on upper-third lesions [31] and undifferentiated-type EGC larger than 2 cm [32,33] suggest that the current thresholds for tumor diameter (size)—3 cm for differentiated-type EGC and 2 cm for undifferentiated-type EGC—seem to require further revision and expansion. Of course, in cases of ulcers, submucosal invasion greater than 500 μm, and undifferentiated-type gastric cancer, the possibility of lymph node metastasis may be higher, indicating the need for the discovery and development of other clinical and histologic factors that can complement and assess these risks.

There are notable differences in the factors influencing the decision-making process between endoscopic and surgical treatment in the esophagus [34,35] and colon [36], where tumor diameter (size) is not considered a risk factor. Unlike other parts of the gastrointestinal tract, it is questionable whether tumor diameter (size) is a significant factor in the stomach. In fact, considering the progression from absolute to expanded indication, it seems that factors that were statistically significant in surgical specimens were taken into account. It is well known that as tumor diameter (size) increases, the tumor tends to be more aggressive (with deeper invasion and a higher likelihood of lymph node metastasis). However, just as we observe superficial-type cancers in the esophagus or laterally spreading tumors in the colon, we can also observe EGCs in the stomach that spread laterally. These types of EGCs can be good candidates for effective endoscopic treatment.

## 4. Conclusions

As endoscopists, we have already faced similar challenges during the transition from absolute indication to expanded indication for endoscopic treatment. Nevertheless, we successfully established expanded indications and accumulated valuable clinical experience. In particular, East Asia, including countries like Japan, China, and Korea, has a high incidence of gastric cancer, a significant number of EGC detections due to screening programs, and a growing emphasis on quality of life. Additionally, with an aging population, there is an increasing number of patients for whom surgery is not a feasible option. A large-scale meta-analysis of elderly patients aged 80 and above who underwent gastrectomy for gastric cancer showed a higher in-hospital mortality rate compared to younger patients (RR, 3.23; 95% CI, 1.46–7.17, *p* < 0.01) [37]. In this context, efforts to narrow the gray zone have become necessary. It seems reasonable, with appropriate caution, for treatment indications to rely more on the outcomes of endoscopic treatment rather than solely on those established from surgical treatment outcomes.

## Figures and Tables

**Table 1:** Evolution of endoscopic treatment indications in the Japanese Gastric Cancer Treatment Guidelines.

	1st (2001)	2nd (2004)	3rd (2010)	4th (2014)	5th (2018)	6th (2021)
D-type, UL(−), T1a and ≤2 cm in diameter	EMR	EMR	Absolute indication (EMR or ESD)	Absolute indication (EMR or ESD)	Absolute indication for EMR or ESD	Absolute indication for EMR or ESD
D-type, UL(−), T1a and >2 cm in diameter	-	-	Expanded indication (not EMR but ESD)	Expanded indication (ESD rather than EMR)	Absolute indication for ESD	Absolute indication for ESD
D-type, UL(+), T1a and ≤3 cm in diameter	-	-	Expanded indication (not EMR but ESD)	Expanded indication (ESD rather than EMR)	Absolute indication for ESD	Absolute indication for ESD
UD-type, UL(−), T1a and ≤2 cm in diameter	-	-	Expanded indication (not EMR but ESD)	Expanded indication (ESD rather than EMR)	Expanded indication	Absolute indication for ESD
Do not fulfill the absolute or expanded indication, option for the elderly and high-operative-risk patients with severe comorbidities	-	-	-	-	Relative indication	Relative indication
A locally recurring lesion after initial endoscopic resection, diagnosed as T1a, for a lesion with an absolute indication and D-type	-	-	Could be treated by another ESD (investigational)	Considered to meet the criteria for expanded indication (investigational)	Considered as expanded indication (need a large-scale observational study)	Expanded indication

D-type: differentiated-type, UD-type: undifferentiated-type, UL: ulceration, EMR: endoscopic mucosal resection, ESD: endoscopic submucosal dissection, T1a: limited to the mucosa.

**Table 2:** The gray zone between endoscopic treatment and surgical treatment.

	Distinct Zone	Gray Zone	Actions or Stages Influencing the Gray Zone	Precautions During Endoscopic Diagnosis and Treatment	The degree of Ambiguity, from the Author’ s Perspectives
Depth of invasion	Intramucosal vs. submucosal cancer (M1, M2, M3 vs. SM) Adequate preservation of the SM layer in the specimen with minimal electrocautery damage to the SM layer	Tumor location (cardia, fundus, body vs. antrum) SM cancer (SM1 vs. SM2 or SM3)	Holes in the specimen or damage to the SM layer	Minimal injury and cauterization of the flap and SM layer	Relatively weak
Histologic Ulceration	No definitive ulceration	Suspicious ulceration, ulcer scar, healing ulcer, and biopsy artifact Effects of medication such as PPIs	Damage caused by biopsy	Reduce the number of biopsies	Relatively strong
Histologic differentiation	Homogeneity	Heterogeneity	-	-	Relatively weak
Tumor diameter	Less than 3 cm in differentiated-type cancer Less than 2 cm in undifferentiated-type cancer	Over 3 cm in differentiated-type cancer (UL, SM1) Over 2 cm in undifferentiated-type cancer	Overstretch of specimen	Minimize curling with appropriate tension, secure with pins, and carefully flatten to avoid overstretching	Strong
Evaluation of Margin	Clear margins of the lesion	Difficulty in identifying the margins of the lesion during histologic diagnosis Burning and laceration of the margin	Marking around the lesion Incision around the lesion	Incision made after ensuring an adequate safety margin around the lesion	Weak
Lymphovascular invasion	Adequate preservation of the SM layer in the specimen with minimal electrocautery damage to the SM layer	Theoretical risk of cellular contamination caused by needle puncture Injury and cauterization of the flap and SM layer	Direct needle puncture of the tumor Direct hemostasis in case of bleeding from the flap	Avoid direct needle puncture of the tumor Hemostasis of perforating vessels instead of flap vessels	Weak

M1: epithelium, M2: lamina propria, M3: muscularis mucosa, SM: submucosa, SM1: the upper third of the submucosa and ≤500 μm, SM2: the middle third of the submucosa, SM3: the lower third of the submucosa, UL: ulceration.

## Data Availability

No new data were created or analyzed in this study. Data sharing is not applicable to this article.

## References

[1] Yamao T., Shirao K., Ono H., Kondo H., Saito D., Yamaguchi H., Sasako M., Sano T., Ochiai A., Yoshida S. (1996). Risk factors for lymph node metastasis from intramucosal gastric carcinoma. Cancer Interdiscip. Int. J. Am. Cancer Soc..

[2] Gotoda T., Yanagisawa A., Sasako M., Ono H., Nakanishi Y., Shimoda T., Kato Y. (2000). Incidence of lymph node metastasis from early gastric cancer: Estimation with a large number of cases at two large centers. Gastric Cancer.

[3] Nakajima T. (2002). Gastric cancer treatment guidelines in Japan. Gastric Cancer.

[4] Japanese Gastric Cancer Association (2004). Gastric Cancer Treatment Guidelines. https://www.jgca.jp/wp-content/uploads/2023/08/Guidelines2004_eng.pdf.

[5] Japanese Gastric Cancer Association (2011). Japanese gastric cancer treatment guidelines 2010 (ver. 3). Gastric Cancer.

[6] Japanese Gastric Cancer Association (2017). Japanese gastric cancer treatment guidelines 2014 (ver. 4). Gastric Cancer.

[7] Japanese Gastric Cancer Association (2021). Japanese gastric cancer treatment guidelines 2018. Gastric Cancer.

[8] Japanese Gastric Cancer Association (2023). Japanese gastric cancer treatment guidelines 2021. Gastric Cancer.

[9] Abdelfatah M.M., Barakat M., Ahmad D., Ibrahim M., Ahmed Y., Kurdi Y., Grimm I.S., Othman M.O. (2019). Long-term outcomes of endoscopic submucosal dissection versus surgery in early gastric cancer: A systematic review and meta-analysis. Eur. J. Gastroenterol. Hepatol..

[10] Kim Y.-I., Kook M.-C., Choi J.E., Lee J.Y., Kim C.G., Eom B.W., Yoon H.M., Ryu K.W., Kim Y.-W., Choi I.J. (2020). Evaluation of submucosal or lymphovascular invasion detection rates in early gastric cancer based on pathology section interval. J. Gastric Cancer.

[11] Yeomans N.D., Naesdal J. (2008). Systematic review: Ulcer definition in NSAID ulcer prevention trials. Aliment. Pharmacol. Ther..

[12] Bereda G. (2022). Peptic Ulcer disease: Definition, pathophysiology, and treatment. J. Biomed. Biol. Sci..

[13] Park Y.S., Kook M.C., Kim B.H., Lee H.S., Kang D.W., Gu M.J., Shin O.R., Choi Y., Lee W., Kim H. (2023). A Standardized Pathology Report for Gastric Cancer: 2nd Edition. J. Gastric Cancer.

[14] SAKITA T. (1973). Endoscopy in the diagnosis of early ulcer cancer. Clin. Gastroenterol..

[15] Joo M.K., Park J.-J., Chun H.J. (2019). Proton pump inhibitor: The dual role in gastric cancer. World J. Gastroenterol..

[16] Myung Y.S., Hong S.J., Han J.P., Park K.W., Ko B.M., Lee M.S. (2017). Effects of administration of a proton pump inhibitor before endoscopic submucosal dissection for differentiated early gastric cancer with ulcer. Gastric Cancer.

[17] Yabuuchi Y., Takizawa K., Kakushima N., Kawata N., Yoshida M., Yamamoto Y., Kishida Y., Ito S., Imai K., Ishiwatari H. (2021). Discrepancy between endoscopic and pathological ulcerative findings in clinical intramucosal early gastric cancer. Gastric Cancer.

[18] Takao M., Kakushima N., Takizawa K., Tanaka M., Yamaguchi Y., Matsubayashi H., Kusafuka K., Ono H. (2012). Discrepancies in histologic diagnoses of early gastric cancer between biopsy and endoscopic mucosal resection specimens. Gastric Cancer.

[19] Shim C.N., Chung H., Park J.C., Lee H., Shin S.K., Lee S.K., Lee Y.C. (2015). Early gastric cancer with mixed histology predominantly of differentiated type is a distinct subtype with different therapeutic outcomes of endoscopic resection. Surg. Endosc..

[20] Yang S., Gu X., Tao R., Huo J., Hu Z., Sun F., Ni J., Wang X. (2022). Relationship between histological mixed-type early gastric cancer and lymph node metastasis: A systematic review and meta-analysis. PLoS ONE.

[21] Ono H., Yao K., Fujishiro M., Oda I., Uedo N., Nimura S., Yahagi N., Iishi H., Oka M., Ajioka Y. (2021). Guidelines for endoscopic submucosal dissection and endoscopic mucosal resection for early gastric cancer. Dig. Endosc..

[22] Çiyiltepe H., Ergin A., Somay A., Esen Bulut N., Fersahoğlu M.M., Köroğlu M., Karip A.B., Akyüz Ü., Memişoğlu K. (2021). The effects of formalin solution on wall thickness and size in stomach resection materials. Bosphorus Med. J..

[23] Choi J., Kim S.G., Im J.P., Kim J.S., Jung H.C. (2013). Endoscopic estimation of tumor size in early gastric cancer. Dig. Dis. Sci..

[24] Shim C.N., Song M.K., Kang D.R., Chung H.S., Park J.C., Lee H., Shin S.K., Lee S.K., Lee Y.C. (2014). Size discrepancy between endoscopic size and pathologic size is not negligible in endoscopic resection for early gastric cancer. Surg. Endosc..

[25] Maducolil J.E., Girgis S., Mustafa M.A., Gittens J., Fok M., Mahapatra S., Vimalachandran D., Jones R. (2024). Risk of tumour seeding in patients with liver lesions undergoing biopsy with or without concurrent ablation: Meta-analysis. BJS Open.

[26] Gao R.-Y., Wu B.-H., Shen X.-Y., Peng T.-L., Li D.-F., Wei C., Yu Z.-C., Luo M.-H., Xiong F., Wang L.-S. (2020). Overlooked risk for needle tract seeding following endoscopic ultrasound-guided minimally invasive tissue acquisition. World J. Gastroenterol..

[27] Kwong W.T.-Y., Coyle W.J., Hasteh F., Peterson M.R., Savides T.J., Krinsky M.L. (2014). Malignant cell contamination may lead to false-positive findings at endosonographic fine needle aspiration for tumor staging. Endoscopy.

[28] Hatta W., Gotoda T., Oyama T., Kawata N., Takahashi A., Yoshifuku Y., Hoteya S., Nakamura K., Hirano M., Esaki M. (2017). Is radical surgery necessary in all patients who do not meet the curative criteria for endoscopic submucosal dissection in early gastric cancer? A multi-center retrospective study in Japan. J. Gastroenterol..

[29] Hatta W., Gotoda T., Oyama T., Kawata N., Takahashi A., Yoshifuku Y., Hoteya S., Nakagawa M., Hirano M., Esaki M. (2017). A scoring system to stratify curability after endoscopic submucosal dissection for early gastric cancer:“eCura system”. Off. J. Am. Coll. Gastroenterol..

[30] Hatta W., Gotoda T., Koike T., Uno K., Asano N., Imatani A., Masamune A. (2022). Is additional gastrectomy required for elderly patients after endoscopic submucosal dissection with endoscopic curability C-2 for early gastric cancer?. Digestion.

[31] Park S.H., Yoon H.M., Ryu K.W., Kim Y.-W., Kook M.-C., Eom B.W. (2022). Risks and benefits of additional surgery for early gastric cancer in the upper third of the stomach meeting non-curative resection criteria after endoscopic submucosal dissection. World J. Surg. Oncol..

[32] Yang H.-J., Nam S.Y., Min B.-H., Ahn J.Y., Jang J.-Y., Kim J., Kim J.-H., Lee W.-S., Lee B.E., Joo M.K. (2021). Clinical outcomes of endoscopic resection for undifferentiated intramucosal early gastric cancer larger than 2 cm. Gastric Cancer.

[33] Bae J.Y., Ryu C.B., Lee M.S., Dua K.S. (2024). Long-term outcomes of endoscopic submucosal dissection for undifferentiated type early gastric cancer over 2 cm with R0 resection. World J. Gastrointest. Endosc..

[34] Obermannová R., Alsina M., Cervantes A., Leong T., Lordick F., Nilsson M., van Grieken N., Vogel A., Smyth E. (2022). Oesophageal cancer: ESMO Clinical Practice Guideline for diagnosis, treatment and follow-up☆. Ann. Oncol..

[35] Kitagawa Y., Ishihara R., Ishikawa H., Ito Y., Oyama T., Oyama T., Kato K., Kato H., Kawakubo H., Kawachi H. (2023). Esophageal cancer practice guidelines 2022 edited by the Japan esophageal society: Part 1. Esophagus.

[36] Hashiguchi Y., Muro K., Saito Y., Ito Y., Ajioka Y., Hamaguchi T., Hasegawa K., Hotta K., Ishida H., Ishiguro M. (2020). Japanese Society for Cancer of the Colon and Rectum (JSCCR) guidelines 2019 for the treatment of colorectal cancer. Int. J. Clin. Oncol..

[37] Merga Z.C., Lee J.S., Gong C.-S. (2023). Outcomes of Gastrectomy for Gastric Cancer in Patients Aged> 80 Years: A Systematic Literature Review and Meta-Analysis. J. Gastric Cancer.

